# Bioresorbable Vascular Stents: How Neutrophil Extracellular Traps Influence Biocompatibility, Degradation Kinetics, and Device Performance

**DOI:** 10.3390/bioengineering13030278

**Published:** 2026-02-27

**Authors:** Rasit Dinc, Nurittin Ardic

**Affiliations:** 1 INVAMED Medical Innovation Institute, New York, NY 10007, USA; 2 Med-International UK Health Agency Ltd., Nuneaton CV11 6LT, Warwickshire, UK; nurittinardic@yahoo.com

**Keywords:** neutrophil extracellular traps, bioresorbable scaffold, biocompatibility, cardiovascular device, immunothrombosis

## Abstract

Bioresorbable scaffolds (BRS; also referred to as bioresorbable vascular scaffolds, BVS) represent a promising approach in interventional cardiology, offering theoretical advantages such as temporary mechanical support followed by complete resorption. However, clinical experience has revealed challenges, including late-stage scaffold thrombosis and heterogeneous scaffold discontinuity during degradation, prompting investigation into host immune responses. Neutrophil extracellular traps (NETs), which are network-like structures composed of decondensed chromatin decorated with antimicrobial proteins, have emerged as critical mediators of vascular inflammation and thrombosis. This review explores the intersection between NET biology and BRS performance, investigating how NETosis affects biocompatibility, degradation kinetics, and device-related complications. We discuss the molecular mechanisms that trigger neutrophil activation and NET formation in scaffold materials, the effect of NET components on polymeric and metallic scaffold degradation, and emerging biomarkers to monitor NET-mediated complications. We also evaluate therapeutic strategies targeting NET pathways, including DNase-based therapies, peptidylarginine deiminase 4 (PAD4) inhibitors, and anti-inflammatory coatings that can optimize next-generation BRS outcomes. Understanding the immunological environment surrounding bioresorbable vascular devices is crucial for developing scaffolds that deliver predictable degradation while minimizing adverse inflammatory responses.

## 1. Introduction

Bioresorbable scaffolds (BRS; also called bioresorbable vascular scaffolds, BVS) were developed to address the limitations of permanent metallic stents, including persistent foreign body response, impaired vasomotor function, and late stent thrombosis [1,2]. The concept of a temporary scaffold that provides initial mechanical support during vessel healing and is then fully absorbed offers theoretical advantages, including restoration of physiological vascular function and elimination of long-term device-related complications [3].

The first-generation Absorb bioresorbable vascular scaffold (Abbott Vascular), composed of poly-L-lactic acid (PLLA) with an everolimus-releasing coating, received regulatory approval based on promising early clinical results [4]. However, long-term follow-up revealed unexpectedly high scaffold thrombosis rates compared to contemporary drug-eluting stents, leading to its withdrawal from the market in 2017 [5,6]. Subsequent analyses identified multiple contributing factors, including scaffold design features (thick struts, limited expansion capacity), implantation technique, and patient selection [7,8]. Importantly, the biological response to the degraded scaffold material emerged as a critical, yet not fully understood determinant of outcomes.

Neutrophil extracellular traps (NETs) have recently been recognized as important mediators of cardiovascular pathology [9,10]. These network-like structures, composed of decondensed chromatin coated with granular proteins including myeloperoxidase (MPO), neutrophil elastase (NE), and cathelicidins, were initially identified as an antimicrobial defense mechanism [11]. However, abnormal or excessive NET formation contributes to thrombosis, inflammation, and tissue damage in various cardiovascular conditions [12,13].

This review investigates how neutrophil activation and NETosis affect scaffold biocompatibility, degradation kinetics, and clinical outcomes by examining the emerging evidence linking NET biology to BRS performance. We discuss potential biomarkers for monitoring NET-related complications and evaluate treatment strategies that could improve next-generation scaffold designs.

This narrative review was conducted following an extensive literature search in PubMed, Scopus, and Web of Science databases between January 2004 and January 2026. Search terms included combinations of ‘bioresorbable scaffold’, ‘bioresorbable vascular scaffold’, ‘bioresorbable stent’, ‘PLLA scaffold’, ‘magnesium scaffold’, ‘neutrophil extracellular traps’, ‘NETs’, ‘NETosis’, ‘immunothrombosis’, and ‘stent thrombosis’. Original English research articles, systematic reviews, and meta-analyses examining scaffold biomaterials, neutrophil biology, NET formation mechanisms, and cardiovascular thrombosis were included. Reference lists of key articles were manually scanned to identify additional relevant publications. Studies were selected based on their relevance to the intersection of NET biology and bioresorbable scaffold performance, and their emphasis on mechanistic information and translational implications.

**Figure 1:**
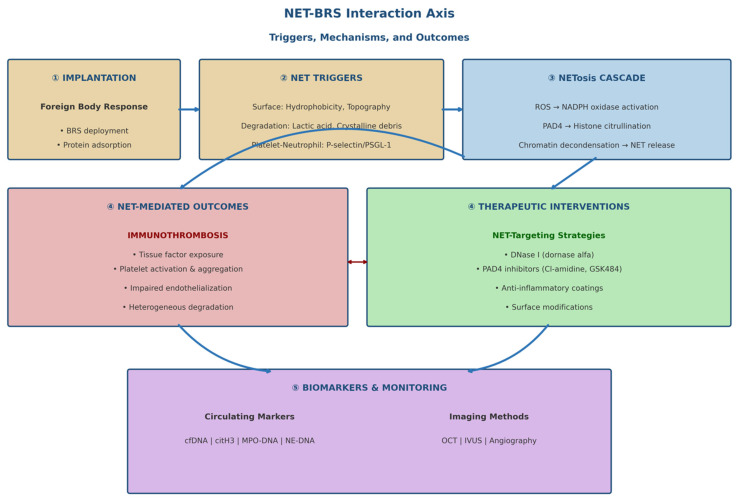
Neutrophil extracellular trap (NET)—bioresorbable scaffold (BRS) interaction axis: triggers, mechanisms, and outcomes. Schematic diagram illustrating the NET-BRS interaction axis. The figure shows: (1) the initial foreign body response following BRS implantation and protein adsorption; (2) NET triggers including scaffold surface properties (hydrophobicity, topography), degradation products (lactic acid, crystalline residues, metal ions), and platelet–neutrophil interactions via the P-selectin/PSGL-1 pathway; (3) the NETosis cascade via ROS production, NADPH oxidase activation, PAD4-mediated histone citrullination, and chromatin decondensation; (4) NET-mediated outcomes including immunothrombosis (tissue factor exposure, platelet activation), impaired endothelialization, heterogeneous degradation, and mechanical instability—along with therapeutic intervention points (DNase, PAD4 inhibitors, anti-inflammatory coatings, surface modifications); (5) circulating biomarkers (cfDNA, citH3, MPO-DNA, NE-DNA) and imaging methods for monitoring.

To provide an integrated overview of the mechanisms discussed in this review, the interactions between bioresorbable scaffolds, neutrophil activation, NET formation, and downstream clinical consequences are schematically summarized in Figure 1.

## 2. Bioresorbable Scaffolds: Current Status and Clinical Challenges

### 2.1. Evolution of Bioresorbable Technology

The development of bioresorbable scaffolds has followed two main material platforms: polymeric scaffolds based on polylactic acid derivatives and metallic scaffolds using magnesium alloys [14]. PLLA-based scaffolds, exemplified by Absorb BVS, produce lactic acid, which enters the Krebs cycle by degrading through hydrolytic cleavage of ester bonds [15]. Complete resorption typically takes 3–4 years, during which time the scaffold transforms from a mechanically supportive structure into an amorphous mass before final clearance [16].

Magnesium-based scaffolds offer faster degradation kinetics compared to PLLA (typically 9–12 months, compared to 3–4 years for PLLA), while iron-based and zinc alloy platforms provide moderate degradation profiles (12–24 months) [17]. The Magmaris scaffold (Biotronik) represents the most advanced clinical version, featuring a magnesium alloy backbone with a bioresorbable polymer coating that elutes sirolimus [18]. Hybrid approaches combining polymeric and metallic components are also being investigated [19]. Table 1 summarizes the main classes of biodegradable exoskeleton materials, their degradation mechanisms, and related immunity and NET considerations.

Zinc alloy scaffolds have emerged as a promising alternative platform offering intermediate degradation rates (typically 12–24 months) between magnesium and polymeric platforms [23]. Zinc exhibits favorable biocompatibility, with Zn^2+^ ions playing essential roles in numerous enzymatic processes and cellular functions. Recently developed Zn-Mg and Zn-Li alloy formulations have demonstrated improved mechanical properties while maintaining suitable corrosion rates for vascular applications [24]. Zinc corrosion products are generally well tolerated, and slower and more homogeneous degradation compared to magnesium can reduce the risk of mechanical failure during the absorption period.

Iron-based scaffolds provide excellent mechanical strength similar to permanent stainless-steel stents, and degradation occurs via oxidative corrosion over 12–24 months [25]. Degradation products (primarily iron oxides) are cleared by macrophages via established cellular iron recycling pathways. However, concerns regarding local iron accumulation, potential inflammatory effects of iron oxide particles, and relatively slow degradation rate necessitate further research [26]. While early clinical experiences with iron-based scaffolds have shown promising results, long-term data are yet to be collected.

#### 2.1.1. Scaffold Fabrication and Characterization Methods

The fabrication of bioabsorbable scaffolds utilizes various techniques depending on the material platform. Polymeric scaffolds are typically produced by extruding polymer tubes, followed by precision laser cutting to create a support pattern [14,15]. Alternative approaches include electrospinning to create fibrous scaffolds and three-dimensional printing using melt deposition modeling or stereolithography for complex geometries [28]. Metallic scaffolds (magnesium, zinc, iron) are produced by casting, extrusion followed by laser cutting, or photochemical etching [17,25].

Surface modification techniques are used to enhance biocompatibility and control drug release. These include plasma treatment, chemical grafting of hydrophilic polymers, and application of drug-delivering coatings [29,30]. Characterization of scaffold materials involves multiple analytical methods: Fourier-transform infrared spectroscopy (FTIR) for chemical composition analysis, nuclear magnetic resonance (NMR) spectroscopy for molecular structure determination, scanning electron microscopy (SEM) for surface morphology and support size assessment, and mechanical tests for radial strength and rebound properties [14,19]. Quality control protocols typically include accelerated degradation tests, biocompatibility tests, and drug release kinetics studies.

#### 2.1.2. Scaffold Absorption Process

The absorption of bioabsorbable scaffolds goes through different stages depending on the type of material. For PLLA-based scaffolds, degradation occurs through hydrolytic cleavage of ester bonds in a four-stage process [15,16]:

Phase 1 (Hydration): Water penetrates the polymer matrix, initiating the degradation process. This phase typically occurs within the first few weeks after implantation.

Phase 2 (Strength Loss): Molecular weight decreases through random chain breakage while maintaining scaffold structural integrity. Radial strength begins to decrease in this phase (3–12 months).

Phase 3 (Mass Loss): Polymer degradation occurs with the formation of oligomers and monomers. Cellular clearance of degradation products begins, and scaffold discontinuity may become apparent in imaging (12–24 months).

Phase 4 (Complete Absorption): Lactic acid is converted to carbon dioxide and water via the Krebs cycle. For PLLA scaffolds, complete absorption usually takes 3–4 years.

Magnesium-based scaffolds undergo corrosion-mediated degradation, releasing magnesium ions and hydrogen gas according to the reaction: Mg + 2H_2_O → Mg(OH)_2_ + H_2_ [17]. The resulting magnesium hydroxide layer provides temporary corrosion protection before gradual dissolution. Complete absorption usually occurs within 9–12 months.

Iron-based scaffolds decompose via oxidative corrosion, subsequently forming iron oxides (mainly Fe_2_O_3_ and Fe_3_O_4_), which are then cleared by macrophages within 12–24 months [25]. Zinc alloy scaffolds exhibit moderate degradation kinetics (12–24 months) via corrosion mechanisms similar to magnesium, but hydrogen release is reduced and degradation patterns are more homogeneous [23,24].

#### 2.1.3. Advantages and Disadvantages of Biologically Absorbable Scaffolds

Biologically absorbable scaffolds offer several theoretical advantages over permanent metallic stents:

(1) Restoration of vasomotor function after scaffold absorption enables physiological vascular responses to vasoactive stimuli and potentially reduces the risk of late-stage adverse events.
(2) Elimination of the persistent foreign body reduces the theoretical risk of very late-stage stent thrombosis and allows for future surgical revascularization options without permanently caged vascular segments.
(3) Facilitates positive vascular remodeling and potential late-stage lumen recovery after scaffold absorption.
(4) Potential to shorten the duration of dual antiplatelet therapy after complete scaffold absorption.
(5) Compatibility with non-invasive imaging methods (MRI, CT) without artifact formation from permanent metallic implants [3,7]. However, significant disadvantages have limited clinical use:
  (1) Thicker support profiles (approximately 150 μm for Absorb, compared to 80 μm for contemporary drug-eluting stents) result in greater flow disturbance and increased thrombogenic potential.
  (2) Lower radial strength compared to metallic stents, requiring careful lesion preparation and precise vessel sizing.
  (3) Long degradation period (3-4 years for PLA), during which thrombotic risk persists and device-related complications may occur.
  (4) Strict vessel sizing protocols, technical requirements during implantation including mandatory pre-dilatation and post-dilatation requirements.
  (5) Higher device cost compared to contemporary metallic stents.
  (6) Higher scaffold thrombosis rates have been observed in clinical trials compared to metallic drug-eluting stents [5,31,32].

### 2.2. Clinical Experience and Complications

The ABSORB III trial (n = 2008), comparing Absorb BVS with Xience everolimus-eluting metallic stents, initially demonstrated equivalent target lesion failure rates at one year [33]. However, extended follow-up revealed significantly higher rates of target lesion failure and device thrombosis with BVS, particularly between one and three years post-implantation [5]. The ABSORB IV trial (n = 2604 patients) and the BIOSOLVE-II trial (n = 123 patients, magnesium scaffold) provided additional safety and efficacy data. Meta-analyses encompassing over 10,000 patients demonstrated that scaffold thrombosis rates were approximately 2–3 times higher than with contemporary drug-eluting stents [31,32].

Several mechanisms have been proposed to explain the high thrombotic risk. Thick scaffold struts (approximately 150 μm for Absorb, compared to 80 μm for current metallic DES) create larger flow disturbances and a greater potential for platelet activation [34]. Scaffold discontinuity during degradation may expose thrombogenic material to the bloodstream [35]. In addition, the inflammatory response to the degradation of the polymer, including neutrophil recruitment and activation, can create a prothrombotic environment that persists throughout the prolonged absorption period [36,37].

## 3. Neutrophil Extracellular Traps: Biology and Cardiovascular Implications

### 3.1. NET Formation Mechanisms

NETosis, the process of NET formation, can occur via multiple pathways with different kinetics and outcomes [38]. The classical pathway, suicide NETosis, involves the formation of reactive oxygen species (ROS) via NADPH oxidase, the transport of granular proteins to the nucleus, and histone citrullination mediated by peptidylarginine deiminase 4 (PAD4) [39]. Citrullination promotes chromatin decondensation by neutralizing histone charges. This is followed by nuclear membrane breakdown, and the rupture of the plasma membrane allows chromatin to mix with cytoplasmic and granular contents before releasing the NET structure [40].

Vital NETosis represents an alternative pathway in which NETs are typically released from living neutrophils via vesicular mechanisms [41]. This process occurs more quickly (while it takes hours for suicidal NETosis, it takes minutes here) and allows neutrophils to retain their antimicrobial functions. The relative contribution of these pathways to biomaterial-induced NETosis remains incompletely characterized and represents an important area for future investigation. Standardized methods for characterizing biomaterial-induced NETosis include the following: flow cytometry-based quantification of citrullinated histone-positive neutrophils; immunofluorescence microscopy for NET visualization using anti-MPO and DNA staining; ELISA-based measurement of NET-specific markers (MPO-DNA complexes, citH3); and real-time impedance-based monitoring of NETosis kinetics [42,43,44]. Consensus protocols for these assays are currently under development to enable cross-study comparisons.

### 3.2. NETs in Cardiovascular Thrombosis

NETs contribute to thrombosis through multiple mechanisms. The DNA backbone provides a scaffold for platelet adhesion and activation, while histones directly activate platelets and damage endothelial cells [45,46]. NET-associated tissue factor initiates the extrinsic coagulation cascade, and NET components, including elastase, increase coagulation activity by inactivating tissue factor pathway inhibitor (TFPI) [47]. Histopathological analysis of thrombus aspirates from patients with stent thrombosis by the PRESTIGE consortium revealed the clinical significance of NET presence in 23% of cases [12]. In acute myocardial infarction, circulating NET markers are associated with infarction size and adverse outcomes [48,49]. NETs have been identified in coronary thrombi, and their presence is associated with impaired microvascular perfusion following percutaneous intervention [50]. These observations suggest that therapies targeting NETs may improve outcomes in acute coronary syndromes and potentially in device-associated thrombosis.

### 3.3. NETs and Endothelial Dysfunction

NET components exert direct cytotoxic effects on endothelial cells. Histones, particularly H3 and H4, cause endothelial cell death through mechanisms involving calcium influx, mitochondrial dysfunction, and inflammatory signal activation [51]. Neutrophil elastase increases vascular permeability by degrading endothelial junction proteins. These effects may be particularly important in the context of BRS, where the endothelial sheath is necessary to isolate thrombogenic scaffold material from the bloodstream and prevent late scaffold thrombosis [51,52].

## 4. Effect of NETosis on Bioresorbable Scaffold Performance

**Figure 2:**
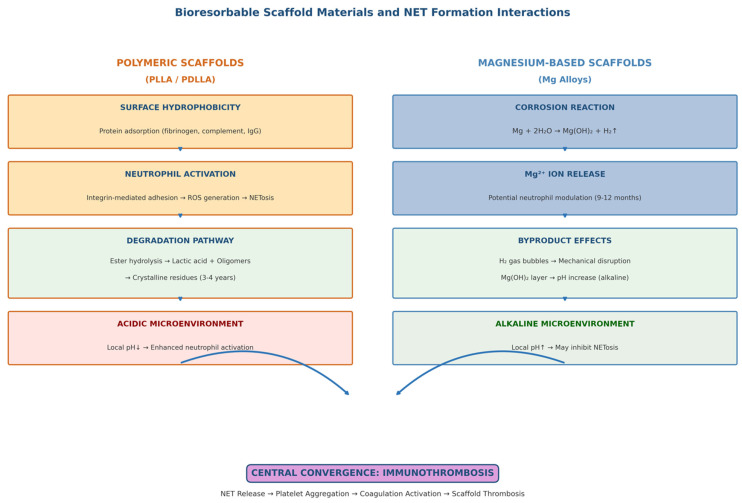
Interactions between bioresorbable scaffold materials and NET formation. Dual-panel schematic comparing immune interactions of polymeric and metallic scaffolds. (**Left** Panel) (Polymeric Scaffolds-PLLA/PDLLA): Surface hydrophobicity → protein adsorption (fibrinogen, complement) → neutrophil adhesion via integrin receptors → ROS generation → NETosis; Degradation pathway: ester hydrolysis → lactic acid + oligomers → crystalline residues → macrophage/neutrophil activation → inflammatory amplification; Acidic microenvironment effects on local pH and neutrophil function. (**Right** Panel) (Magnesium-Based Scaffolds): Corrosion reaction (Mg + 2H_2_O → Mg(OH)_2_ + H_2_) → Mg^2+^ ion release → potential neutrophil modulation; Hydrogen gas bubble formation → mechanical disruption; Hydroxide layer formation → pH increase → alkaline microenvironment effects. Central Convergence: Both pathways lead to NET release, platelet aggregation, coagulation activation, and immunothrombosis.

Since the composition of the scaffold material critically determines local immune responses, Figure 2 illustrates the different ways in which polymeric and magnesium-based bioabsorbable scaffolds interact with neutrophils and promote NET formation.

### 4.1. Biomaterial-Induced NET Formation

The interaction between neutrophils and biomaterial surfaces is governed by protein adsorption, surface topography, and chemical properties. Upon contact with blood, scaffolds rapidly acquire a protein corona dominated by fibrinogen, complement components, and immunoglobulins [21,53,54]. These adsorbed proteins facilitate neutrophil adhesion via integrin receptors and trigger activation pathways that can lead to NETosis [55,56].

Biomaterial surface properties, including hydrophobicity and topography, can promote neutrophil activation and NET formation, which is important for PLA-based scaffold platforms [57]. Importantly, Xie et al. demonstrated that modification of PLA with carbon monoxide (CO)-loaded iron-based metal–organic frameworks (FeMOFs) significantly reduced NET release by decreasing intracellular ROS production [6]. This suggests that scaffold material properties can be designed to modulate the NET response. Scaffold degradation products can provide additional NETosis triggers. Lactic acid accumulation during PLLA hydrolysis creates an acidic microenvironment that can enhance neutrophil activation [4,15,20]. Crystalline degradation intermediates formed by the conversion of the amorphous polymer into crystalline residues can function as particle triggers, such as monosodium urate or cholesterol crystals [58]. For magnesium scaffolds, hydrogen gas formation during corrosion causes mechanical degradation, while the released magnesium ions can modulate neutrophil function through effects on intracellular signaling [22].

### 4.2. Effects of NETs on Scaffold Degradation Kinetics

NET components can directly influence scaffold degradation through enzymatic and chemical mechanisms. Neutrophil elastases and other proteases released during NETosis can accelerate polymer chain breakage, potentially creating heterogeneous degradation patterns [59]. The acidic pH created through respiratory burst activity by active neutrophils can locally enhance the hydrolytic degradation of polyester-based scaffolds [60]. For magnesium scaffolds, chloride ions in NET-associated DNA can accelerate corrosion by disrupting the protective surface layers [61]. Conversely, the alkaline environment created by the formation of magnesium hydroxide during corrosion can theoretically neutralize acidic conditions that support neutrophil activation, thereby inhibiting NETosis. These complex interactions between scaffold chemistry and NET biology are not yet fully characterized and represent important areas for future research.

### 4.3. NETs as Mediators of Scaffold Thrombosis

The prothrombotic properties of NETs are particularly concerning in the context of BRS, where thrombotic complications represent the primary clinical limitation. NETs can integrate scaffold struts and form a matrix that supports platelet aggregation and fibrin formation [62]. Histone-mediated endothelial damage can delay the protective endothelial sheath necessary for scaffold integration and prolong the thrombotic fragility window [63].

The temporal relationship between scaffold disruption and NET-mediated thrombotic risk deserves special attention. Clinical data show that the risk of BRS thrombosis peaks during the degradation phase, when scaffold discontinuity exposes the inner surfaces and releases degradation products [64]. This timing coincides with sustained inflammatory activity surrounding the degrading device, suggesting a mechanistic link between inflammation-induced NETosis and late thrombotic events [65,66].

## 5. Biomarkers for Monitoring NET-Mediated Complications

### 5.1. Circulating NET Biomarkers

Extracellular DNA (cfDNA) represents the most easily measurable component of NETs, as NETs release significant amounts of extracellular DNA during their formation. High plasma cfDNA levels have been associated with adverse outcomes in acute coronary syndromes and correlate with infarct severity [67,68,69]. However, cfDNA is not specific to NETosis, as it can also arise from other forms of cell death, including apoptosis and necrosis. More specific NET markers include citrullinated histones (particularly citrullinated H3 or citH3) produced during NETosis, particularly via PAD4 activity [39,67]. MPO-DNA complexes formed when myeloperoxidase remains associated with extracellular DNA provide another relatively specific marker of NET formation [68].

Neutrophil elastase-DNA complexes (NE-DNA) and calprotectin have also been investigated as NET biomarkers. Recent studies have shown that composite biomarker panels containing multiple NET markers can provide superior prognostic information compared to individual markers [68]. The development of standardized assays for determining NET quantification remains an active area of research, with ELISA-based methods, flow cytometry, and imaging approaches all being investigated [42,43,44].

### 5.2. Imaging Approaches for NET Detection

Non-invasive imaging of NET accumulation in scaffold regions represents an attractive but technically challenging goal. Intravascular imaging methods, including optical coherence tomography (OCT) and intravascular ultrasound (IVUS), can assess scaffold integrity and thrombus presence but cannot specifically identify NET components [70]. Research efforts are exploring molecular imaging approaches that can visualize NET-specific targets in vivo, but these remain at the experimental stage [71].

## 6. Therapeutic Strategies Targeting NET Pathways

Table 2 summarizes the key NET-inducing mechanisms, relevant biomarkers, and potential therapeutic modulation strategies related to biodegradable scaffolds.

### 6.1. DNase-Based Therapies

Deoxyribonuclease (DNase) enzymes, which degrade extracellular DNA, offer a direct approach to NET clearance. DNase I, available as recombinant dornase alfa (Pulmozyme), has been extensively studied for its NET-degrading properties. Animal studies have shown that DNase therapy reduces thrombus formation and improves outcomes in myocardial infarction models. Improved DNase variants with enhanced stability and activity are under development [72,73,74].

### 6.2. PAD4 Inhibitors

Peptidylarginine deiminase 4 (PAD4), the enzyme responsible for histone citrullination during NETosis, represents an attractive therapeutic target. Mice deficient in PAD4 show reduced NET formation and protection from thrombosis in various models [75]. Small-molecule PAD4 inhibitors have been developed and have shown efficacy in preclinical studies [76,77]. These agents could theoretically prevent NET formation while preserving other neutrophil functions and potentially offer a better safety profile than complete neutrophil depletion.

Recent studies have aimed to achieve localized NETosis inhibition while minimizing systemic effects by investigating nanoparticle-mediated delivery of PAD4 inhibitors to sites of vascular damage [78]. This approach may be particularly important for scaffold applications, where inflammation is concentrated at the device–tissue interface. However, the clinical development of PAD4 inhibitors is in its early stages, and their safety in the context of cardiovascular disease requires careful evaluation.

### 6.3. Anti-Inflammatory Approaches

Several established anti-inflammatory agents have shown effects on NET formation. Colchicine, which inhibits microtubule polymerization and neutrophil activation, has been shown to reduce NET formation in vitro and improve outcomes after myocardial infarction in the COLCOT study [79,80]. Metformin, commonly used in diabetes treatment, reduces NETosis through AMPK-dependent mechanisms [81]. Although statins are sometimes reported to increase NET formation, they can also exhibit protective effects through pleiotropic anti-inflammatory effects [82,83].

### 6.4. Surface Engineering Approaches

Modification of scaffold surfaces to reduce NETogenic potential offers a device-centric approach to limiting NET-mediated complications. Strategies investigated include hydrophilic coatings that reduce protein adsorption and neutrophil activation [29], phosphorylcholine-based zwitterionic surfaces that mimic cell membrane chemistry [30], and controlled release of anti-inflammatory agents from the scaffold surface. The aforementioned FeMOF/CO system represents an innovative approach that combines surface modification with therapeutic gas delivery [6]. Carbon monoxide, at controlled low concentrations, exhibits anti-inflammatory effects by reducing ROS production and inhibiting NETosis. Similar strategies involving nitric oxide donors or other anti-inflammatory molecules can further enhance the biocompatibility of the scaffold [84]. The challenge is to ensure sustained release during the long-term degradation period while maintaining the mechanical integrity of the scaffold.

## 7. Current Gaps and Methodological Limitations

Despite the increasing acceptance of the role of NETs in BRS complications, several critical deficiencies limit our current understanding and clinical practice:

• Lack of standardized NET assays: There is no consensus on optimal methods for determining NET quantification in clinical samples; variable sensitivity and specificity across different test platforms limit comparability between studies.
• Limited human pathology data: Few studies have directly examined the presence and burden of NETs in relation to BRS impairment status. Much of the evidence linking NETs to stent complications is inferential or derived from samples of metallic stent thrombosis.
• Confounding factors: The contribution of dual antiplatelet therapy (DAPT) intensity, implantation technique, lesion complexity, and patient-specific inflammatory profiles makes it difficult to isolate the impact of NET-specific effects on stent outcomes.
• Animal model limitations: Current animal models do not fully replicate human coronary flow dynamics, atherosclerotic plaque composition, or the chronic inflammatory environment present in patients with coronary artery disease.
• Lack of direct NETogenicity comparisons: Systematic comparisons of the NET-inducing potential among different BRS materials (PLLA, PCL, magnesium alloys) and surface modifications are lacking.

Addressing these shortcomings will require coordinated multicenter biobanking studies, the development of consensus testing protocols, and the integration of NET assessments into future BRS clinical trials.

## 8. Future Directions

**Figure 3:**
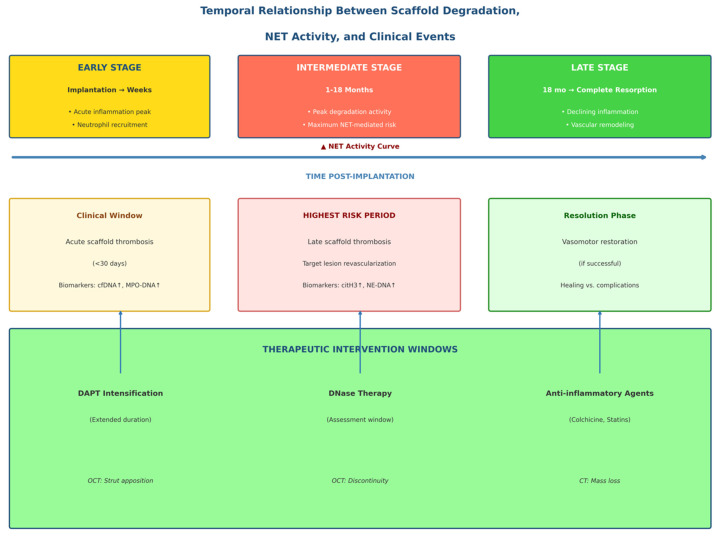
Temporal relationship between scaffold deterioration, NET activity, and clinical events. Timeline-style diagram showing the three phases of scaffold–tissue interaction. Early Stage (implantation → weeks): Acute inflammation peak; neutrophil recruitment due to procedural trauma; initial protein adsorption and foreign body response; elevation in basal NET activity; clinical window for acute scaffold thrombosis (<30 days). Biomarker overlap: elevated cfDNA, MPO-DNA. Imaging window: OCT for strut apposition. Intermediate Stage (1–18 months): Peak degradation activity; sustained inflammatory cell infiltration; maximum NET-mediated thrombogenic risk; heterogeneous scaffold discontinuity; bulk erosion of polymer matrix. Biomarker overlap: citH3, NE-DNA complexes. Clinical events: late scaffold thrombosis, target lesion revascularization. Late Stage (18 months → complete resorption): Declining inflammation; Progressive scaffold mass loss; vascular remodeling; restoration of vasomotor function (if successful). Clinical outcome: healing vs. persistent complications. The treatment intervention timeline indicates optimal time intervals for DAPT intensification, DNase therapy assessment, anti-inflammatory agents, surface modification effects.

To place NET-mediated processes within a clinically meaningful temporal framework, Figure 3 illustrates the evolving relationship between scaffold degradation, NET activity, biomarker dynamics, and device-related clinical events at different stages post-implantation.

The integration of NET biology into BRS development opens up various avenues for future research and device optimization. Next-generation scaffold design should incorporate immunological considerations in addition to traditional mechanical and chemical optimization. This may involve systematically screening the NETogenic potential of candidate materials during preclinical development using standardized in vitro tests and appropriate animal models [85,86].

Reducing support thickness through improved materials and fabrication techniques can reduce inflammatory stimulation while maintaining mechanical performance [87]. Advances in layered fabrication allow for complex geometries that can distribute mechanical stress more homogeneously and reduce localized inflammation [28]. Hybrid scaffold designs combining the advantages of different materials, such as metallic cores and polymeric coatings, offer additional possibilities to optimize both mechanical and biological performance [27].

Personalized approaches to scaffold selection and adjuvant therapy present another frontier. Patients with high baseline NET markers or genetic variants affecting NETosis pathways may benefit from intensified anti-inflammatory therapy or alternative revascularization strategies. The development of point-of-care NET tests can enable real-time risk stratification and treatment optimization.

Finally, lessons learned from the BRS development process regarding immune responses to degradable materials have implications beyond cardiovascular devices. Similar principles apply to other implantable medical devices, tissue engineering scaffolds, and drug delivery systems. A deeper understanding of NET–biomaterial interactions can shed light on the design of next-generation implantable devices in multiple therapeutic areas [88]. To highlight the environmental significance of NET biology for scaffold therapy, Table 3 summarizes NET-related clinical complications, potential monitoring strategies, and the current state of clinical evidence.

## 9. Conclusions

The interaction between neutrophil extracellular traps and bioresorbable scaffolds represents a significant and understudied aspect of device biocompatibility. Evidence increasingly suggests that NETosis contributes to scaffold-related complications, including thrombosis, delayed healing, and heterogeneous degradation. Understanding these immunological mechanisms is essential for the development of next-generation scaffolds that deliver on the promise of transient mechanical support followed by complete, seamless absorption. Therapeutic strategies targeting NET pathways, including DNase enzymes, PAD4 inhibitors, and anti-inflammatory agents, offer potential approaches to mitigate NET-mediated complications. Device-centric strategies focusing on surface engineering and material selection can reduce the NETogenic potential of scaffolds at the source. The optimal approach likely combines both systemic and local interventions tailored to individual patient risk profiles.

Future research should prioritize the standardization of NET measurement methodologies, the systematic characterization of NET–material interactions for candidate scaffold materials, and the integration of NET biomarkers into clinical trial outcome points. Collaboration among immunologists, materials scientists, and interventional cardiologists will be essential to translate these findings into improved clinical outcomes for patients receiving bioresorbable vascular scaffolds.

## Figures and Tables

**Table 1:** Bioresorbable Scaffold Materials and Immuno–Degradation Properties.

Material Type	Degradation Mechanism	Manufacturing Methods	Immune Interaction Profile	Potential NET-Associated Risks	Key References
PLLA/PDLLA	Hydrolytic ester bond cleavage; absorption in 3-4 years; produces lactic acid	Extrusion, laser cutting, 3D printing	Surface hydrophobicity promotes protein adsorption; acidic microenvironment increases neutrophil activation	Crystalline debris triggers NETosis; prolonged inflammation window; late thrombogenic events	[4,14,15,16,20]
Magnesium alloys	Corrosion; absorption in 9–12 months; releases Mg^2+^ ions and hydrogen gas	Casting, extrusion, laser cutting	Mg^2+^ can regulate neutrophil function; hydrogen bubbles cause mechanical disruption	Rapid corrosion can overwhelm local clearance; effects of ions on NET formation are unclear	[17,18,21,22]
Zinc alloys	Corrosion; absorption in 12–24 months; releases Zn^2+^ ions	Casting, extrusion, electroforming	Zn^2+^ has immunomodulatory effects; moderate and homogeneous corrosion rate	Limited NETogenicity data available; requires systematic evaluation	[23,24]
Iron-based	Oxidative corrosion; absorption in 12–24 months; forms iron oxides	Electroforming, powder metallurgy, 3D printing	Iron oxide particles are cleared by macrophages; potential for local inflammation	NET interactions under investigation; iron accumulation concerns	[25,26]
PCL	Slower hydrolysis than PLA; absorption in 2–3 years	Electrospinning, 3D printing, solvent casting	More hydrophobic surface; different protein corona formation	Less studied in terms of NETogenicity; potentially lower acute inflammation	[14,19]
Hybrid/Composite	Combined polymer–metal; variable degradation kinetics	Layer-by-layer, co-extrusion, coating	Complex interface effects; multiple degradation product types	Potential for synergistic or antagonistic immune effects; requires systematic evaluation	[19,27]

PLLA, poly-L-lactic acid; PDLLA, poly-D,L-lactic acid; PCL, polycaprolactone.

**Table 2:** NET triggers, biomarkers, and therapeutic modulation strategies in bioresorbable scaffolds.

Category	NET-Related Mechanism	Biomarkers	Intervention Strategy	Evidence Level	Key References
Material surface	Hydrophobicity → protein adsorption → integrin-mediated neutrophil activation → ROS → NETosis	Serum cfDNA, MPO-DNA complexes	Hydrophilic coatings; zwitterionic surfaces; PC-modified polymers	In vitro/Animal	[29,30,57]
Degradation products	Lactic acid → acidic pH → increased neutrophil activation; crystalline residues → particle-induced NETosis	Local pH sensors; lactate levels	Buffering agents; controlled degradation kinetics; amorphous formulations	In vitro/Animal	[20,58,60]
Platelet–neutrophil crosstalk	P-selectin/PSGL-1 binding → reciprocal activation → platelet-induced NETosis	Platelet–neutrophil aggregates; soluble P-selectin	Extended DAPT; P-selectin inhibitors; GPIIb/IIIa antagonists	Clinical (DAPT); preclinical (others)	[45,46,47]
Enzymatic clearance	NET DNA backbone provides matrix for thrombosis; histone cytotoxicity	cfDNA; citH3; NE-DNA complexes	DNase I (dornase alfa); enhanced DNase variants	Animal/Early clinical	[72,73,74]
PAD4 pathway	PAD4-mediated histone citrullination → chromatin decondensation → NET release	Citrullinated H3 (citH3)	PAD4 inhibitors (Cl-amidine, GSK484); nanoparticle-targeted delivery	In vitro/Animal	[75,76,77,78]
Anti-inflammatory	General neutrophil activation and recruitment → NETosis amplification	CRP; IL-6; neutrophil counts	Colchicine; metformin; statins (variable effects); anti-IL-1β	Clinical (colchicine, statins); preclinical (others)	[79,80,81,82,83]

**Table 3:** Clinical Effects and Monitoring Strategies Associated with NET in Scaffold Therapy.

Clinical Issue	NET Involvement	Biomarkers	Imaging Modality	Translational Status	Key References
Scaffold thrombosis (early/late)	NETs provide a prothrombotic scaffold; histone-mediated platelet activation; tissue factor exposure	cfDNA, MPO-DNA, citH3	OCT (thrombus); angiography	Clinical observations established; biomarker validation ongoing	[12,45,46,62]
Delayed endothelialization	Histone cytotoxicity to endothelial cells; NET-mediated barrier dysfunction	Circulating endothelial cells; endothelial microparticles	OCT (strut coverage)	Mechanistic evidence in vitro; clinical correlation limited	[51,52,63]
Heterogeneous degradation	Elastase-mediated polymer degradation; acidic pH resulting from respiratory burst	Scaffold integrity markers (investigational)	OCT (discontinuity); CT (mass loss)	Preclinical evidence; clinical studies needed	[59,60,61]
Neoatherosclerosis	NET-induced inflammation promotes lipid accumulation and plaque formation	Lipid markers; inflammatory cytokines	OCT (lipid-rich plaque); NIRS	Emerging concept; long-term studies needed	[64,65]
In-scaffold restenosis	NET-associated growth factors promote smooth muscle proliferation	PDGF; TGF-β levels	Angiography; OCT; FFR	Indirect evidence; mechanistic studies ongoing	[36,37]

## Data Availability

No new data were created or analyzed in this study.
